# HEARING LOSS AND PHYSICAL ACTIVITY AMONG OLDER ADULTS IN THE UNITED STATES

**DOI:** 10.1093/geroni/igae098.1074

**Published:** 2024-12-31

**Authors:** Sahar Assi, Erica Twardzik, Jennifer Deal, Kathleen Martin Ginis, Priya Palta, Jennifer Schrack, Nicholas Reed, Pablo Martinez Amezcua

**Affiliations:** Johns Hopkins University, Baltimore, Maryland, United States; University of Michigan, Ann Arbor, Maryland, United States; Johns Hopkins University, Baltimore, Maryland, United States; University of British Columbia Okanagan, Kelowna, British Columbia, Canada; University of North Carolina, Chapel Hill, North Carolina, United States; Johns Hopkins Bloomberg School of Public Health, Baltimore, Maryland, United States; Johns Hopkins University, Baltimore, Maryland, United States; Johns Hopkins University, Baltimore, Maryland, United States

## Abstract

**Background:**

Hearing loss is associated with adverse health outcomes among older adults, which may be partly explained by lower physical activity levels. Yet, the association between hearing loss and physical activity among older adults remains understudied.

**Methods:**

Cross-sectional analysis of the National Health and Aging Trends Study (2021). The better-hearing ear pure-tone average (BPTA) at speech frequencies (0.5-4 kHz) was modeled continuously (per 10-dB) and categorically (no: ≤25 dB, mild: 26-40 dB, moderate or greater: >40 dB hearing loss). Activity measures were 1) wrist accelerometry-derived total activity counts, daily active minutes, and activity fragmentation, and 2) self-reported participation in vigorous activities and walking for exercise in the last month.

**Results:**

Among 504 participants excluding hearing aid users (mean age = 79 years, 57% female, 9% Black), 338 (67%) had hearing loss. Using multivariable regression adjusted for sociodemographic and health covariates, we found that worse hearing (continuously and categorically) was associated with fewer counts and active minutes, more fragmented activity, and greater odds of not reporting recent vigorous activities. Among 472 participants with hearing loss, nonusers of hearing aids (n = 338) had more fragmented activity and greater odds of not reporting walking for exercise compared to users. Conclusions: Older adults with hearing loss are less physically active. This may mediate the association between hearing loss and other adverse outcomes. Recognition of this potential association is essential for providers to better support older adults in maintaining an active lifestyle. Future research is warranted to understand the impact of hearing interventions.

